# Doxycycline Prevents Intimal Hyperplasia In Vitro and May Improve Patency of the Internal Thoracic Artery

**DOI:** 10.1155/2013/217026

**Published:** 2013-08-22

**Authors:** Vito Mannacio, Luigi Di Tommaso, Anita Antignano, Ettorino Di Tommaso, Paolo Stassano, Carlo Vosa

**Affiliations:** ^1^Department of Cardiac Surgery, School of Medicine, University Federico II, Via S. Pansini 5, 80131 Naples, Italy; ^2^Department of Cardiology, Azienda Ospedaliera Santobono-Pausilipon, Via Posillipo 226, 80123 Naples, Italy

## Abstract

*Objectives*. The development of intimal hyperplasia and graft failure is an important problem in cardiac surgery. A fundamental process in intimal hyperplasia is the degradation of extracellular matrix by metalloproteases which induces the vascular smooth-muscle cells migration and sets the scene for graft atherosclerosis. This study investigated whether doxycycline, a metalloproteases inhibitor, can prevent the intimal hyperplasia occurrence in cultured human internal mammary artery, thus extending graft patency. *Methods*. Segments of internal mammary artery from 20 consecutive patients were prepared and cultured for 2 weeks in serum-supplemented medium (control) or in medium supplemented with 10^−5^ M and 10^−6^ M doxycycline concentrations. Tissues were fixed, sectioned, and stained, and neointimal thickness was measured by computer-aided image analysis. Further sections were cultured and prepared for gel enzymography to measure the matrix metalloproteinase-2 and -9 levels. *Results*. At the end of the culture period, neointimal thickness was significantly (*P* = 0.001) dose-dependently reduced in samples treated with doxycycline when compared with controls. Gelatin enzymography demonstrated a reduction in values for both latent and active forms of metalloproteases. *Conclusions*. Doxycycline, in a model of internal mammary artery intimal hyperplasia, has a specific role in inhibiting metalloproteases activity and may prevent graft stenosis.

## 1. Introduction

Intimal hyperplasia is the pathologic hallmark of the occlusion of graft after a myocardial revascularization procedure, and, although more frequent in venous grafts, it does not spare the internal mammary artery (IMA) [1–3].

 Intimal hyperplasia is characterized by migration of vascular smooth-muscle cells (VSMCs) through the internal elastic lamina into the arterial intima and subsequent proliferation to produce the intimal hyperplastic lesion [4]. VSMCs are able to produce and secrete the necessary proteases to degrade all components of the extracellular matrix (ECM) which is a prerequisite to VSMCs migration [4, 5]. This probably involves altered expression, production, and regulation of the matrix metalloproteinase proteins (MMPs), mainly gelatinase A (MMP-2) and gelatinase B (MMP-9), which break down the basement membrane permitting cell migration [5]. In addition to the possible role of impaired expression of nitric oxide, enhanced activity of MMPs has been thought to increase the development of atherosclerosis [6, 7]. Therefore, MMPs enzymes provide an attractive target for pharmacologic agents aimed at reducing the development of intimal hyperplastic lesion and the development of atherosclerotic lesions.

Doxycycline is one of these pharmacologic agents. It is a member of the tetracycline family of antibiotics and is known to exert biological effects that are independent of its antimicrobial activity. Doxycycline reduces both gelatinase MMP-2 and MMP-9 expression and activity by restoring the balance between matrix degradation and deposition and shows good side-effect profiles even if used for many months or years [8]. Doxycycline has been evaluated over the time for the experimental treatment of pathological conditions involving MMPs, such as aortic aneurysms [9], myocardial infarction [10], and cancer [11]. Nevertheless, although the effect of doxycycline on MMP-2 and MMP-9 expression and activity has been well assessed, it has not been investigated hitherto in the human IMA intimal hyperplasia.

The aim of this study was to investigate the role of MMP-2 and MMP-9 in the production of arterial intimal hyperplasia and its inhibition by doxycycline in an in vitro model of human arterial intimal hyperplasia.

## 2. Material and Methods

### 2.1. Population of Study

Patient's written consent was obtained, and the local ethics committee approved the protocol. Segments of the IMA were obtained from 20 consecutive patients undergoing off-pump myocardial revascularization at our institution. Their clinical data are detailed in Table 1.

The IMAs were harvested pedicled together with veins and surrounding tissue as previously described [12, 13]. Low-voltage (20 mV) cautery was always used, and the side branches were closed by metal clips.

### 2.2. Culture Methods

Surgical protocol for LIMA segments collection was similar in all patients. All specimens were obtained at the distal end (1 cm proximally and 0.5 cm after bifurcation) immediately after harvesting and before any hydrostatic distension or intraluminal papaverine injection to avoid possible endothelial damage. Surrounding adventitial and fat tissues were carefully removed, and the specimens were divided into 3 segments of approximately 5 mm length. Each segment was opened longitudinally and put, with the endothelial side up, in Petri plate cell-culture disk into which a 5 mm layer of Sylgard 184 encapsulating resin (Dow Corning, Seneffe, Belgium) was cast so as to form a central well. The well was the correct size to accept the silicone rubber support. Organ cultures were maintained in tissue culture medium RPMI 1640 supplemented with 30% calf serum, antibiotics (streptomycin 0.1 mg/mL and penicillin 100 U/mL) and L-glutamine 2 mmol/L. The medium was changed every 48 hours, and at each change glutamine was added. The cultures were maintained at 37°C and gassed with humidified 5% CO_2_ in air. One culture served as control. The other two cultures were supplemented with doxycycline at 10^−5^ M and 10^−6^ M concentrations, obtained with a concentration of doxycycline of 5 mg/L and 0.5 mg/L, respectively. Drug was renewed at each medium change. We used half the dosage reported in the literature to test the efficacy of doxycycline even at half dosage to lessen collateral effects [14]. Drug was renewed at each medium change. After 14-day culture period, IMA specimens were fixed in 4% paraformaldehyde solution for 24 hours, paraffin-embedded and then sectioned in a microtome to ~5 *μ*m thickness. Tissue sections were mounted on APES (amino-propyl-tri-ethoxy-silane) coated slides.

### 2.3. Immunohistochemistry

Immunohistochemistry was performed with deparaffinised, ethanol-rehydrated tissue sections. In order to identify the layers of the arterial wall, sections of ~5 *μ*m thickness were incubated for 15 hours at 4°C with antialpha smooth-muscle actin antibody (Abcam, Cambridge, UK). This was followed by application of Miller's elastin stain to localize the internal elastic lamina. An average of 10 measurements of neointimal thickness was made on each of 2 consecutive sections of each sample using a computer-aided image analysis system. Lining layer thickness was measured by drawing perpendicular lines from the outer surface to the inner margin of the lining layer at intervals of 20–30 *μ*m. These lines were then measured and the mean result was expressed in microns.

### 2.4. Gelatin Zymography

A further 10 samples from consecutive patients were prepared in the same manner as reported above, and after the 14-day period of culture each specimen was immediately frozen and stored at −80°C in liquid nitrogen for subsequent processing and MMP-2 and MMP-9 extraction. Secretion of MMP-2 and MMP-9 was analyzed by gelatine zymography following the method indicated by Porter and colleagues [15]. The frozen samples were defrosted over ice, weighed, cut into pieces of ~1 mm^2^, and then homogenised in 1 mL of buffer per 1 mg of tissue. The homogenate was then centrifuged at 10,000 g for 60 minutes at 4°C, and the supernatant dialysed against a dialysing buffer for 18 hours at 4°C. Zymography was performed on each sample with nonreducing electrophoresis through a 10% sodium dodecyl sulfate-polyacrylamide gel impregnated with 1 mg/mL gelatin. On completion of the separation sodium, dodecyl sulfate was removed from the gels. Gels then were fixed and stained in 0.1% Coomassie Brilliant Blue G250 in 50% methanol/20% acetic acid/30% double distilled water for 2 to 3 hours. Conditioned medium from HT-1080 cells (a fibrosarcoma cell line that constitutively expresses MMP-2 and MMP-9) was loaded on each gel as a positive control. Gelatinases were visualized as clear bands of lysis against a dark blue background of intact substrate. All images were digitalized, and densitometric analysis was performed on negative images. The product of the optical density and area of the band was compared to the H 1080 control.

### 2.5. Statistics

Measurements of neointimal thickness are expressed as median and 95% CI. Differences between treatment groups were analyzed using the Wilcoxon paired-rank test, and significance was assumed at the 95% confidence level. For gelatine zymography analysis, the relative density was quantified and compared between samples run on the same gel. Densitometric values are expressed as a percentage of control (HT 1080 cell line). Values are expressed as median and 95% CI as a percentage of HT 1080 control value. Differences between groups were analyzed with the Wilcoxon test. These analyses were performed with SPSS 8.0 package for Windows.

## 3. Results

### 3.1. Neointimal Thickness

**Figure 1:**
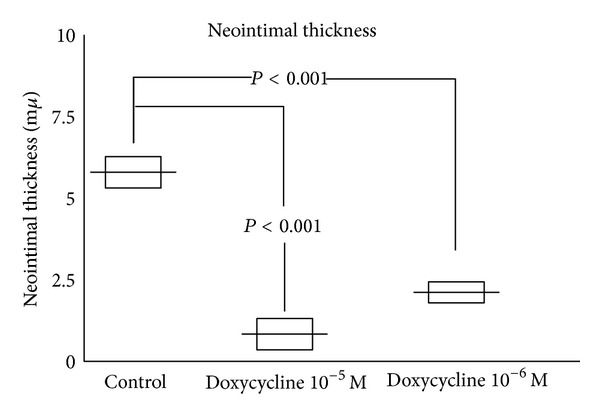
Measurements of neointimal thickness in patient's internal thoracic arteries treated with doxycycline and in controls. Horizontal bars indicate median. Boxes reflect the 95% confidence intervals.

**Figure 2:**
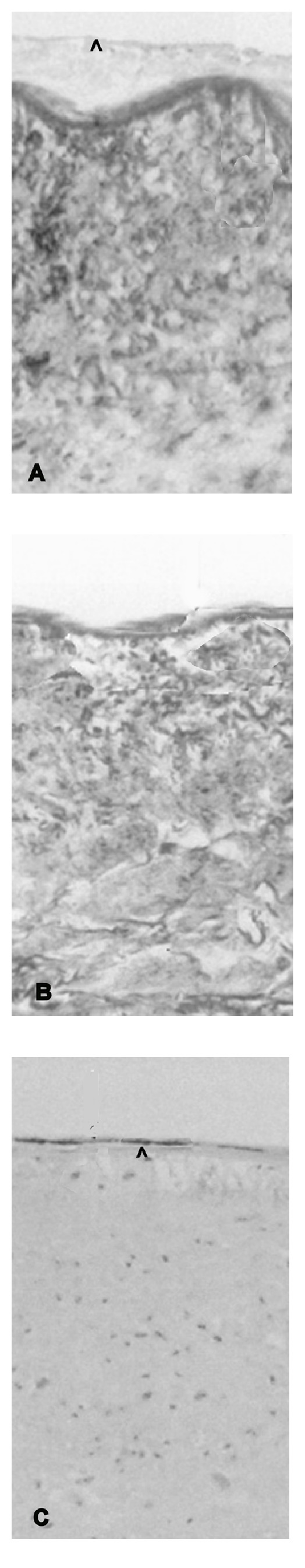
Histologic sections of cultured internal thoracic arteries showing the development of neointima (arrow) in segments from control (A) and doxycycline-treated arteries (B). Combined smooth-muscle actin and elastin stain. The endothelium is intact (C) at the end of the 2-week period of culture (arrow).

At the end of the 2-week period of culture, all the control IMA developed a neointimal layer with a median thickness of 5.9 ± 0.8 *μ*m (95% CI 5.5–6.3). Specimens of IMA treated with doxycycline at 10^−5^ M showed a median neointimal layer of 0.81 ± 0.15 *μ*m thickness (95% CI 0.75–0.87) with a *P* < 0.001, while at 10^−6^ M the neointimal thickness was 1.9 ± 0.3 *μ*m (95% CI 1.8–2.2) with a *P* < 0.001, Figure 1. A section of IMA treated and not treated with doxycycline is depicted in Figure 2. All the IMA segments retained an intact endothelium Figure 2.

### 3.2. Gelatin Zymography

**Figure 3:**
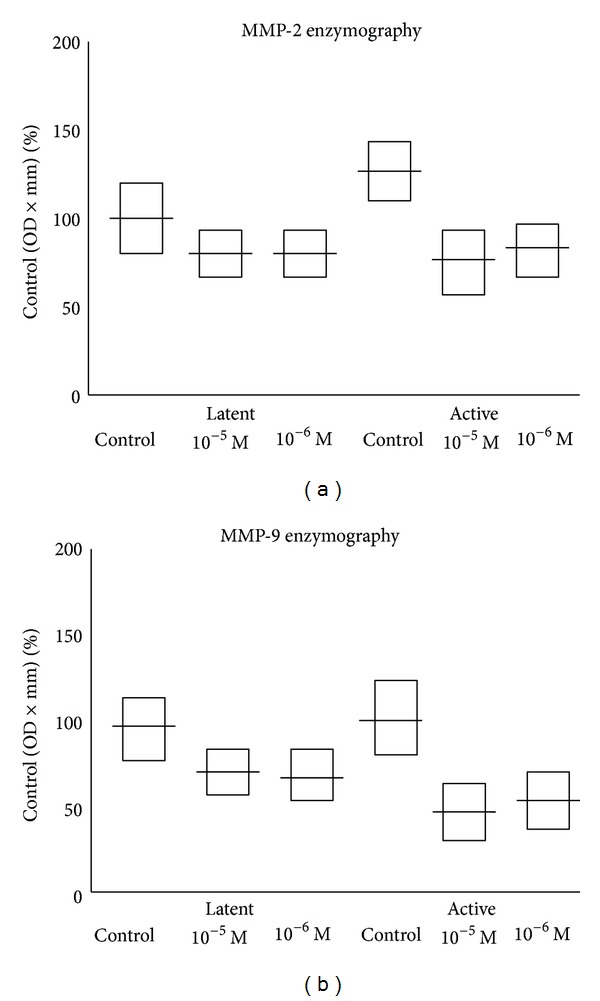
Measurements of MMP-2 (a) and MMP-9 (b) activity of cultured internal thoracic arteries. Horizontal bars represent the median and 95% confidence intervals.

Densitometric values are expressed as a percentage of control (HT 1080 cell line). In the control group of IMA, the densitometric median value for latent MMP-9 was 99% ± 20 (95% CI 86–112), while in the group of IMA treated with doxycycline at 10^−5^ M and at 10^−6^ M the median values were, respectively, 69% ± 17 (95% Cl 58–80) and 66% ± 21 (95% Cl 53–79), significantly lower as in Figure 3. For active MMP-9 in the control group, the median value was 101% ± 32 (95% CI 81–121) while in the group of IMA treated with doxycycline at 10^−5^ M and at 10^−6^ M the median values, respectively, were 43% ± 14 (95% Cl 34–52) and 48% ± 12 (95% Cl 41–55), significantly lower as in Figure 3. For latent MMP-2 in the control group the median densitometric value was 100% ± 21 (95% CI 87–113), while in the group of IMA treated with doxycycline at 10^−5^ M and at 10^−6^ M, the median values, respectively, were 80% ± 15 (Cl 95% 71–89) and 78% ± 17 (95% Cl 68–88), significantly lower as in Figure 3. For active MMP-2, in the control group, the median value was 120% ± 25 (95% CI 105–145), while in the group of IMA treated with doxycycline at 10^−5^ M and at 10^−6^ M, the median values, respectively, were 70% ± 23 (95% Cl 56–84) and 76% ± 17 (95% Cl 65–87), significantly lower as in Figure 3.

**Figure 4:**
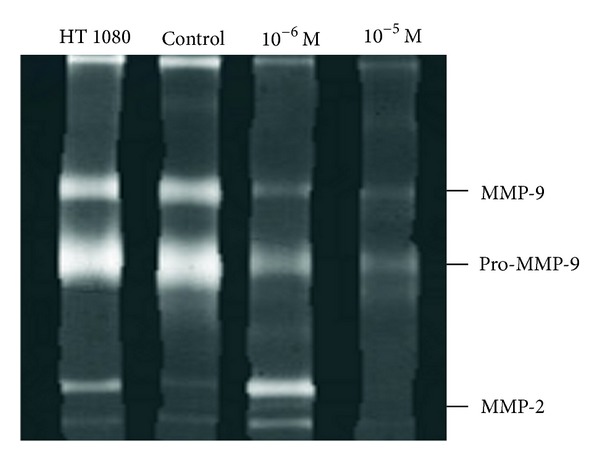
Paired gelatin zymograms for control and doxycycline-treated internal thoracic artery segments. Fibrosarcoma derived HT-1080 cells as positive control.

Gelatin zymogram, both for segment of control IMA and for IMA cultured with doxycycline at 10^−5^ M and at 10^−6^ M, is represented in Figure 4. A representative gelatine zymogram (Figure 4) of the IMA segments treated both with and without doxycycline demonstrated that these samples had less MMP-2 and MMP-9 activity when compared with those cultured in medium (molecular weight range 60–100 kDa).

## 4. Discussion

This study demonstrates that doxycycline significantly reduces the formation of neointima in a laboratory model of IMA. This reduction in the thickness of the neointima is also accompanied by a reduction in the tissue levels of MMP-2 and MMP-9.

Intimal hyperplasia is a disease that occurs early after grafting predisposing the arteries to later accelerated atherosclerosis, and, although elastic arteries such as the IMA are less prone to the development of intimal hyperplastic lesion, they nevertheless are not completely free. Ojha et al., in an histologic study of several IMA grafts, found that the distal anastomosis develops significantly more intimal hyperplasia than the graft body and in time progression lesion at the anastomosis could lead to IMA graft failure [2]. Mekontso-Dessap et al. cultured rings of human saphenous vein, radial artery, and IMA with fetal calf serum and induced intimal hyperplastic lesion development in all 3 types of vessels used for CABG surgery [16]. Furthermore, it has also been demonstrated that intimal hyperplasia not only occurs after the grafting, as we usually know, but also could be already present at the time of operation in arterial conduits [1]. Mild to moderate intimal hyperplasia of the IMA at the time of operation has been detected to a variable degree ranging between 22.5% and 68.7% [3, 17]. This preexisting intimal hyperplasia in the IMA may be an indicator for the systemic nature of arteriosclerosis thus affecting graft disease and postoperative results.

The link between preoperation graft intimal hyperplasia and postoperation graft atherosclerosis is still unclear. So far studies in stenosed and occluded arterial and venous bypass grafts demonstrate that intimal hyperplasia represents an accelerated stage of graft disease, leading subsequently to stenosis [1, 18]. Moreover, excessive lipoproteins in the plasma tend to accumulate preferentially in the hyperplastic intima, causing atherosclerosis [3]. The management of intimal hyperplasia is therefore critical to preserve graft function, and the necessity for alternative strategies has suggested that potential therapeutic agents should be specifically targeted to the pathophysiologic process within the arterial wall.

Matrix metalloproteases are required for intimal hyperplasia development, mainly because of their effects on VSMC migration. Therefore limiting the MMP-2 and MMP-9 activity with pharmacologic agents may prevent VSMC migration and intimal hyperplasia development also in arterial graft, thus extending their performance.

Increased levels of MMP-2 and MMP-9 have been demonstrated in pig arterial vessels damaged by balloon angioplasty [19]. The inhibition of MMPs, while reducing the MMPs activity, did not affect the neointimal formation [20]. In our study MMP-2 and MMP-9 reduction was also accompanied by a reduction of neointima formation. Similar findings were observed by Peterson in a model of human arterial hyperplasia with the use of Marimastat, and he speculated that the human arterial lesion is less cellular and contains more ECM than the lesion in arterial rat model [4]. Similarly, Li et al. studied the effect of GM6001, an MMPs inhibitor, and demonstrated reduction of VSMC migration and neointimal hyperplasia in injured arterial segments [21].

Doxycycline belongs to the family of tetracyclines. These groups of antibiotics are frequently used in relatively low doses for months or years treatments, have good side-effect profiles, and have proven long-term safety and efficacy. Doxycycline nonselectively inhibits MMPs by binding to the active zinc sites and also by binding to an inactive calcium site, which causes conformational change and loss of enzymatic activity [14]. Secondary mechanisms of inhibition have also been proposed, which include a reduction in activation, decreased gene expression, and stabilization of specific and nonspecific inhibition [18]. Clinical trials have also demonstrated that a brief period of doxycycline treatment has a selective effect on vascular inflammation in the abdominal aortic aneurysm [20]. Indeed doxycycline has been shown to forestall abdominal aortic aneurism formation and growth in various animal models of the disease [8, 22].

Based on the pattern and extent of gelatinase activity and the response to the MMP inhibitor doxycycline, our results suggest that MMPs play a critical role in intimal hyperplasia formation and that doxycycline has a significant inhibitory effect in a model of internal thoracic artery hyperplasia.

### 4.1. Limitations

An important feature of atherosclerosis is its segmental quality. Therefore, the extent of disease in one section of a vessel does not necessarily define the condition of the entire vessel. The only solution to this problem is to section serially the entire length of the artery and this was not possible.

Experiments were performed under static culture conditions and so do not take into account variables encountered in the in vivo situation, such as blood flow, pressure, and differences in the architecture of the artery wall.

## 5. Conclusion

The management of intimal hyperplasia with its late potential consequences is currently one of the critical problems in cardiac surgery, and it is clearly desirable that pharmacologic treatments are developed to retard the formation rate of such lesion. This study suggests that doxycycline may be an interesting agent in slowing the rate of occurrence of intimal hyperplasia thus preserving ITA function. The therapeutic potential of this family of drugs in preventing graft failure deserves further consideration.

## Figures and Tables

**Table 1:** Clinical profile of patients.

Variables	*n* = 20 pts
Prior smoking history *n* (%)	14 (70)
Cholesterol level >250 mg/dL *n* (%)	10 (50)
Hypertension *n* (%)	12 (60)
Positive family history *n* (%)	9 (45)
Diabetes *n* (%)	7 (35)
Creatinine plasma level >2.5 mg/dL *n* (%)	1 (0.5)
Peripheral arteries atherosclerosis *n* (%)	2 (10)
